# Blue Light from Cell Phones Can Cause Chronic Retinal Light Injury: The Evidence from a Clinical Observational Study and a SD Rat Model

**DOI:** 10.1155/2021/3236892

**Published:** 2021-05-16

**Authors:** Huili Li, Ming Zhang, Dahong Wang, Guojun Dong, Zhiwei Chen, Suilin Li, Xiaohong Sun, Min Zeng, Haiyang Liao, Huifang Chen, Shengyan Xiao, Xiaodan Li

**Affiliations:** ^1^Department of Ophthalmology, Chongqing Hospital of Traditional Chinese Medicine, Chongqing 400021, China; ^2^Department of Ophthalmology, Huaxi Hospital, Sichuan University, Chengdu 610041, China; ^3^Department of Pathology, Chongqing Hospital of Traditional Chinese Medicine, Chongqing 400021, China; ^4^School of Optoelectronic Engineering, Chongqing University, Chongqing 400044, China; ^5^Department of Radiology, Chongqing University Cancer Hospital & Chongqing Cancer, Institute & Chongqing Cancer Hospital, Chongqing 400030, China

## Abstract

**Background:**

To investigate the chronic photodamage induced by the low-intensity blue light of phones, we carried out a clinical pilot study and established an animal model by irradiating SD rats with a homemade illuminator.

**Methods:**

Clinical investigation: A total of 25 clinical medical workers in our hospital were selected and divided into a control group and an observation group according to the daily video terminal use time. Multifocal electrophysiological system (Mf-ERG) was used for retinal functional examination. Animal experiment: A total of sixty SD rats were randomly divided into a control group (*n* = 6) and an experimental group (*n* = 54). The experimental rats were divided into nine groups, which were exposed to the blue light illuminator of the simulated cell phone array for different time. The visual electrophysiology of the rats was tested, and changes in structure were observed by H&E staining and transmission electron microscopy.

**Results:**

In clinical investigation, macular centers near the concave area retinal photoreceptor cells have reduced amplitude. In animal experiments, the amplitude of photoreceptor cells decreased, the peak time was delayed, and the amplitudes were lower in the experimental groups. H&E staining and transmission electron microscope showed retinal tissue structure and functional damage in experimental groups.

**Conclusions:**

Long-term exposure to low-illuminance blue light can cause retinal tissue structure and functional damage, and the chronic damage due to low-illuminance light warrants attention. The clinical registration number is 2018-KY-KS-LHL.

## 1. Introduction

As we know, the blue light of the screens of mobile phones and computers is harmful to people [1]. Blue light is a light that has always existed in nature, and blue light is safe within a certain range. However, if the human eye is exposed to high frequency and high energy blue light for a long time, retinal damage is inevitable. Blue light is also known as “high energy visible light”, referring to wavelengths of 400 to 500 nm, and only short-wavelength blue light is damaging [2] [3].

LED light sources contain different doses of shortwave blue light (400 nm-480 nm), which widely exists in lighting equipment, computers, mobile phones, and other electronic products [4, 5]. Previous studies revealed that exposure to blue light for a long time can cause retinal light damage [6]. At present, research on blue light is mainly focused on acute light injury with high illuminance (>1000 lux) [4, 7, 8]. However, research regarding the effect of low-illuminance (<100 lux) blue light on the retinal tissue structure remains scarce, and most of the experiments used irradiation by a blue light point source [9–11]. The irradiance of a point light source will disperse with increasing irradiating distance [12]. However, an area array light source has a large irradiance range in the same plane, the irradiance of the light source at the same distance from the vertical center is less different, the irradiance is more uniform, and the experimental data are more stable [13, 14].

Blue light irradiation increases the reactive oxygen species in the retina. Excessive reactive oxygen species causes abnormal oxygen metabolism in the cells, forming severe oxidative stress and destroying the body's normal redox dynamic balance. Oxidative stress caused lipofuscin deposition in retinal pigment epithelium cells, drusen, and choroidal microvascular changes, which ultimately promoted the occurrence of age-related macular degeneration (AMD) [15].

To evaluate the effect of early macular visual function objectively, noninvasively, and quantitatively, the response of several small retinal regions in the stimulated macular area was studied. Based on the clinical data survey results, we found that prolonged use of a video terminal will affect the retina. Then, based on the extensive use of cell phones in daily life in all the user groups, we used animal models to simulate cell phone area array blue light for further exploration. Based on the above information, the current study is aimed at exploring the role of low-illuminance blue light in retinal tissue structure and functional damage using irradiated SD rats with simulated blue light from a cell phone array.

## 2. Methods

### 2.1. Subjects

**Figure 1:**
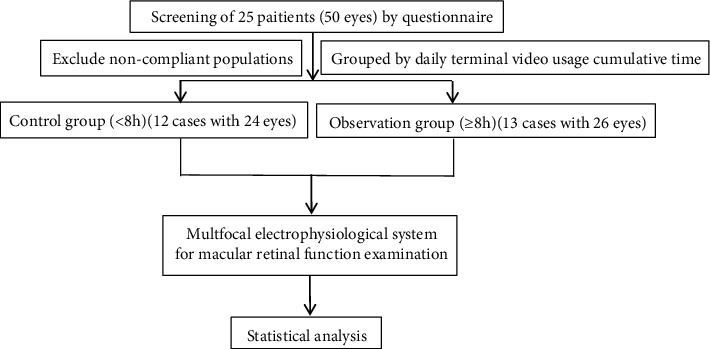
An overview of the clinical pilot study protocol.

Ethical approval and written informed consent were obtained for this study. The human and animal experiments in this research were approved by the ethical committee at Chongqing Hospital of Traditional Chinese Medicine. Twenty-five long-term VDT operation volunteers (50 eyes) who worked in our hospital from 2018 to 2019 were selected. Volunteers were between 24 and 36 years old, with an average age of 28.86 ± 3.50 years. Diopter ≤ −3.00 D (astigmatism < −1.00 D, half of the astigmatism was included in the equivalent spherical power). The total video duration of using mobile phones, computers, and other terminals was >5 years. Those with eye disease or who were pregnant or breastfeeding were excluded. This study was approved by the Chongqing Hospital of Traditional Chinese Medicine. The clinical investigation inclusion criteria and process are shown in Figure 1. There were 3 males (12.00%) and 22 females (88.00%). The right eye was the dominant eye in 19 cases (79.17%), and the left eye was the dominant eye in 6 cases (20.83%). The survey file for the daily use of video terminals is shown in supplementary material 1.

### 2.2. Animals

All rats were provided by the experimental animal center of Chongqing Medical University. SPF (specific pathogen free) SD rats were raised in separate cages at room temperature (18-20°C), a relative humidity of 40%-70%, and a ventilation of 8-12 times/h under a 12/12 h light/dark cycle. A total of sixty (male-female ratio, 1 : 1) 6-week-old SD rats were randomly divided into a normal control group (*n* = 6) and an experimental group (*n* = 54). The normal control group was fed without any intervention. The experimental group was divided into nine groups (3 h, 6 h, and 12 h, tested at 4 weeks, 8 weeks, and 12 weeks). Each day, the rats were irradiated with a simulated cell phone array blue light illuminator (455 nm-470 nm, 5.03 lux (Ee = Ev/Km/V), Ee = 0.123 W/m^2^) for 3 h (08 : 00-11 : 00), 6 h (08 : 00-14 : 00), or 12 h (08 : 00-20 : 00), respectively. From 20 : 00 to the next day 08 : 00 was dark adapted time. Visual electrophysiology was performed at 4 weeks, 8 weeks, or 12 weeks. Then, samples were collected. The breeding and use of laboratory animals followed the Use of Animals in Ophthalmic and Vision Research statement.

### 2.3. Multifocal Electroretinogram Examination

All inspections are performed by the same technician. A German Rolland reti-port/scan 21 multifocal electrophysiological system was used. The stimulator was 19 color graphic stimulators with a LED background screen, the frame frequency was 50~60 Hz, and the stimulus field was 61 hexagons with gradually increasing eccentricity, occupying the eye. The horizontal diameter of the bottom image was 53.2°, while the vertical diameter was 44.2°. Each hexagon had a pseudorandomly assigned black and white inversion. The maximum brightness was 220 CD/m^2^, the minimum brightness was 3 CD/m^2^, and a diagonal “X” shape was set in the center of the stimulus field. The signal was amplified 150,000 times, with the high frequency ending at 100 Hz and the low frequency ending at 10 Hz.

### 2.4. The Manufacture of Blue Light Illuminator from an Analog Cell Phone Array

**Figure 2:**
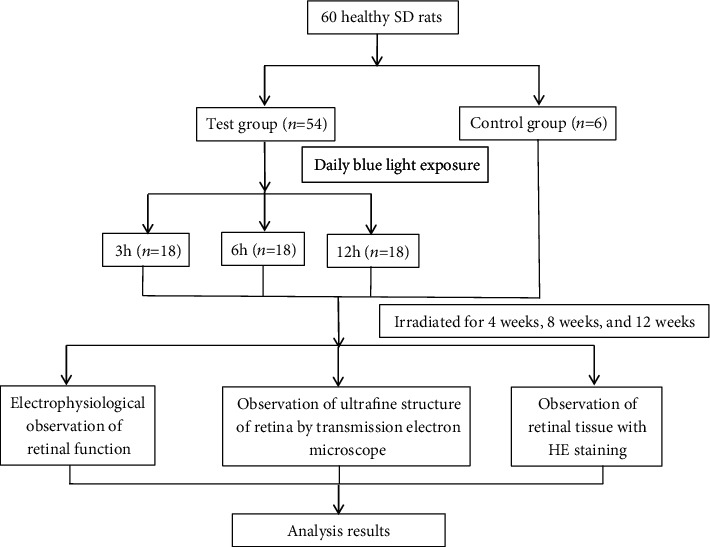
An overview of the experimental protocol.

The spectrum of the white screen of the mobile phone is composite light. Light other than blue light will increase the blue light illuminance. The blue light illuminance value of the mobile phone is equal to the value measured after the mobile phone composite light passes through the filter divided by the light transmittance of the filter. In a dark room, the calibrated illuminance meter equipped with the light-transmitting lens with a wavelength of only 435-465 nm required for this experiment was placed vertically 33 cm from the mobile phone. The filter was parallel to and covered the detection head, and the mobile phone position was measured in the same horizontal plane to measure the brightest value of the mobile phone. According to the measurement results (Table 1), the illuminance of the brand D after being filtered by the filter at a distance of 33 cm from the mobile phone was 2.83 lux, and the light transmittance of the filter, as calculated previously, was 56.27%. The blue light illuminance of the mobile phone was calculated to be 2.83/0.5627 = 5.03 lux (0.123 W/m^2^). An overview of the experimental protocol is shown in Figure 2.

**Figure 3:**
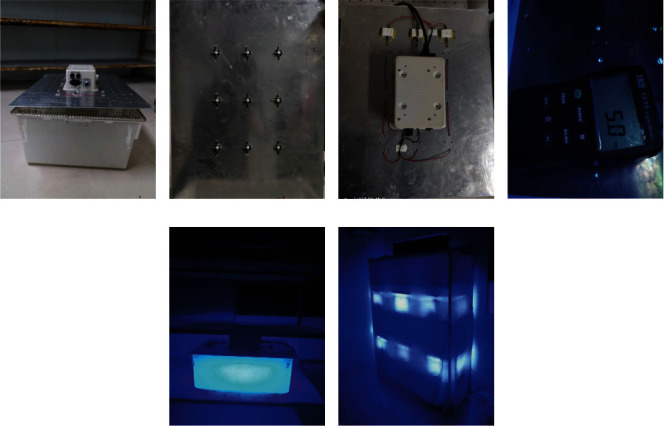
Introduction of the blue light illuminator model for the cell phone array. (a–c) Polyethylene PP feeding cage, with nine blue light bead illuminators on the top cover. (d) The illumination measured by the zd-10 illuminance meter at the four corners and at the midpoint of the four sides of the experimental box bottom 20 cm away from the illuminator light source was 5.0-5.2 lux, with an average illumination of 5.0 lux and a fluctuation range of 5%. (e, f) The model simulating the blue light illumination of cell phones.

Based on the blue light illuminance test of several mainstream mobile phones on the market, the mobile phone with the lowest blue light illuminance was selected as the experimental mobile phone, and the white screen was set to the maximum brightness to generate the white screen spectral radiation. The blue light wavelength of the experimental mobile phone was between 435 nm and 465 nm, and the peak was 449 nm, as detected by the Chongqing Medical Equipment Quality Inspection Center. Because the white screen spectrum is a composite spectrum, the blue illuminance cannot be directly measured, and a qb4 colored glass filter that can pass through the blue light wavelength was selected in this experiment to filter out the nonblue light spectrum. Considering that the standardized reading distance was 33 cm, the blue illuminance value at 33 cm from the illuminator was measured as 480.5 lux. The blue illuminance value measured after the qb4 filter was 270.4 lux with a light transmittance of 56.27% (270.4/480.5). After adding the qb4 filter, the blue light illuminance value of the mobile phone was measured to be 2.83 lux. According to the above calculation, the light transmittance of the qb4 filter was 56.27%, and the actual blue light illuminance value of the experimental mobile phone was identified as 5.03 lux (2.83/0.5627, 0.123 W/m^2^).  Figure 3 introduces the blue light illuminator model for the cell phone array.

### 2.5. Electrophysiological Test

On the day before the electrophysiological test, SD rats were dark adapted for more than 12 h, 10% chloral hydrate injection was anesthetized according to 0.35 ml/100 g anesthesia volume of SD rats, compound tropicamide eye drops were fully mydriatic, 0.4% obucaine hydrochloride eye drops were topically anesthetized, SD rats were fixed on the Espion visual electrophysiological animal experimental platform, and electrodes were placed under dark red light. Electroretinogram (ERG+OPS) detection: the Espion light-guided special corneal electrode was placed on the surface of the cornea of SD rats, with the largest contact between the electrode and the cornea of rats; the silver needle reference electrode was placed under the skin between the eyes of SD rats, and the silver needle ground electrode was placed under the skin of the tail root of rats. Record the time frequency of 1 Hz, space frequency of 0.1 cycle/degree, contrast of 99%, passband of 5-30 Hz, and rod response (rod-r).

### 2.6. Hematoxylin and Eosin (H&E) Staining

The rats were killed by rapid cervical dislocation, and the eyeballs were removed immediately, put into a AR analysis solution of pure acetone (50 ml), glacial acetic acid (2 ml), 40% formaldehyde (4 ml), and distilled water (3 ml), and fixed for 24 h. After 2 h, a small window was opened at the corneal edge of the eyeball specimen to facilitate the infiltration of the fixation solution. The eyeball specimen was fixed for another 2 days, removed and immersed in the following solutions: 80% alcohol for 4 h, 95% alcohol for 4 h of fine immersion, and then 100% alcohol for 4 h. Then, dehydration, fixation, and xylene fixation were performed. After three hours of wax immersion, embedding, sagittal sectioning at a thickness of 4 *μ*m, and routine H&E staining, optical microscopy was performed to observe the changes in retinal tissue structure.

### 2.7. Transmission Electron Microscopy (TEM) Analysis

Rat eyeballs were extracted by the same method and placed in a flat plate on ice. The anterior segment of the eyes was quickly removed by a sharp blade. The posterior segment of the remaining eye samples was fixed in 4°C 2.5% glutaraldehyde solution for 2 h. The retina at the back of the equator was removed, and a 1.5 mm × 4 mm tissue sample was cut and placed in fresh 4°C 2.5% glutaraldehyde solution for full fixation. The sample was washed with phosphate buffer (0.1 M PBS), fixed with 1% osmium acid for 2 h, rinsed with 0.1 M PBS, sequentially dehydrated with 50% and 70% acetone, dyed with 70% acetone uranium overnight at 4°C, sequentially dehydrated with 90% acetone and 100% acetone, and embedded in epoxy resin. The sections were 1 *μ*m thick, and light microscopy was used. After azure methylene blue staining, 70 nm ultrathin sections were stained with conventional lead citrate. Ultrastructural changes in the retina were observed by transmission electron microscopy.

### 2.8. Statistical Analysis

The measurement data are represented as *x* ± *s*, and the count data were represented by the number of cases and composition ratio. For continuous data, a normality test was first performed. If each group met the normality criteria, and if the variances between the two groups were homogeneous, a *t*-test was used for intergroup comparisons, and a paired *t*-test was used to compare the left and right eyes. If the above conditions are not satisfied, a nonparametric test and a paired rank sum test were used. *P* < 0.05 was considered statistically significant. The SPSS 24.0 statistical software was used to analyze the variance different treatment methods and different radiation doses with a multivariate factorial design between. The test method was the Wilks' Lambda method. Based on the interaction of treatment methods and radiation doses, the multiple mean vectors of each group were compared at each time level, and the Hotelling *T*^2^ test was used.

## 3. Results

### 3.1. Mf-ERG Showed Blue Light from the Terminal Video for a Long Time Induced Macular Center near the Concave Area Functional Damage

In the observation group, there were 13 patients (26 eyes), 3 males (23.08%) and 10 females (76.92%). The average age was 27.92 ± 3.35 years. The dominant eye was the right eye in 10 cases (76.92%), and in 3 cases (23.08%), the dominant eye was the left eye. The control group included 12 female patients (24 eyes). The average age was 28.83 ± 3.74 years. The dominant eye was the right eye in 9 cases (75.00%) and the left eye in 3 cases (25.00%). Diopter ≤ −3.00 D. The half quantity of dispersion is included in the equivalent sphericity (SE). There was no significant difference in gender, age, or dominant eye between the two groups (*P* > 0.05). In the comparison of amplitude density and peak time of the N1 and P1 waves in each ring of Mf-ERG, there was no significant difference between and within the two groups (*P* > 0.05). Amplitude density and peak time of N1 and P1 waves in the Mf-ERG 2 ring between the two groups showed a statistically significant difference (*P* < 0.05). The paired *t*-test of the left and right eye data showed that the difference in the P1 wave peak between the left and right eyes was statistically significant in the observation group (*P* < 0.05) (Table 2). In the left-right eye matching *t*-test, a statistically significant difference in the P1 peak between the left and right eyes was found in the observation group (*P* < 0.05) (Table 2). Macular centers near the concave area retinal photoreceptor cells have reduced amplitude, and the peak latency uncovered the fact that blue light from the terminal video watched for a long time may cause macular center near the concave area function in retinal photoreceptor cells decreasing. All clinical tests are not less than 6 times.

### 3.2. ERG Showed Low-Illumination Blue Light Induced Retinal Functional Damage in Rats

To further explore the mechanism of long-term low-illumination blue light damage to the retina, we established an animal model by irradiating SD rats with a homemade illuminator. In the rats' visual electrophysiological examination, the amplitude of the A and B waves in the experimental group was lower than that in the normal control group at different doses (3 h, 6 h, and 12 h), and the incubation period was longer. There was a significant difference in the interaction of different irradiation times and doses (*P* < 0.0001). The amplitudes of wave A and wave B decreased with increasing irradiation dose, and the latency prolonged with increasing irradiation dose in the same irradiation time group. The decline in the electrophysiological function of the retina in the experimental group was significantly positively correlated with the irradiation dose (*P* < 0.0001) (Tables 3–6). These tests were repeated 3 times for each rat, and there were 6 rats per group.

The test method used was the Wilks' Lambda method. *Λ* is the Wilks statistic, indicating the proportion of each variation source in the total variation (*F* is the *F*-test statistic value, *ν*1 is the degree of freedom of the processing factor, and *ν*2 is the degree of freedom of the error term). These tests were repeated 3 times for each rat, and there were 6 rats per group.

### 3.3. H&E Showed Blue Light Induced Retinal Morphological Changes of Rat Eyes

**Figure 4:**
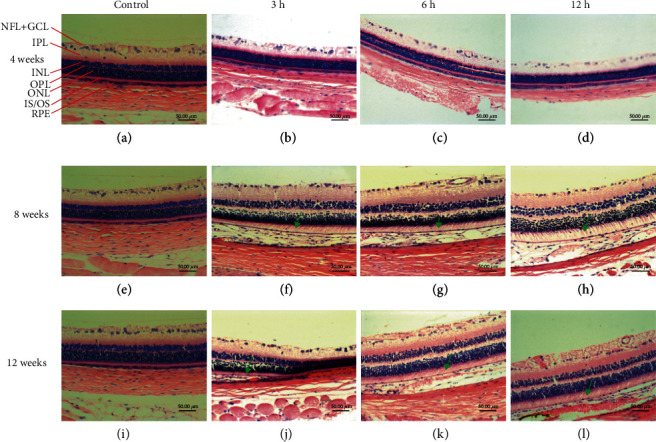
Histopathological features of the retina in each group. (a–d) The structure and morphology of the retina in the 4-week control group and daily exposure of 3 h, 6 h, and 12 h experimental groups. There was no significant change in the IS/OS and RPE layer structure of the retina in the 3 h, 6 h, and 12 h experimental groups. (e–h) The structure and morphology of the retina in the 8-week control group and 3 h, 6 h, and 12 h experimental groups. The IS/OS layer was loose and edematous (green arrow) at 3 h, 6 h, and 12 h after 8 weeks with thinning of the RPE layer and the decrease of the number of cells. (i–l) The structure and morphology of the retina in the 12-week normal control group and 3 h, 6 h, and 12 h experimental groups. The IS/OS layer was loose and edematous (green arrow) lightly stained and swollen (green arrow) with thinning of the RPE layer and the decrease of the number of cells. The scale bar is 50 *μ*m. These tests were repeated 3 times for each rat, and there were 6 rats per group. NFL: nerve fiber layer; GCL: ganglion cell layer; IPL: inner plexiform layer; INL: inner nuclear layer; OPL: outer plexiform layer; ONL: outer nuclear layer; IS/OS: photoreceptor inner/outer segment layers; RPE: retina pigmented epithelium.

As shown in Figures 4(a), 4(e), and 4(i), the retina structures of the control rats at 4, 8, and 12 weeks were intact, and the loose connective tissues were observed. The rats in experimental groups were continuously irradiated for 4 weeks, and there were no significant changes in the IS/OS and RPE layer structure of the retina in the daily exposure of 3 h, 6 h, and 12 h groups (Figures 4(b)–4(d)). And the rats in experimental groups were continuously irradiated for 8 weeks, at daily exposure of 3 h and 6 h, and the IS/OS layers were loose and edematous (green arrow) in some areas with lightly stained, thinning of the RPE layer and the decrease of the number of cells (Figures 4(f) and 4(g)). In the daily exposure of 12 h group, the IS/OS layer was further loosened and edematous, and the cytoplasm was transparent with thinning of the RPE layer and the decrease of the number of cells (Figure 4(h)). After 12 weeks of continuous irradiation, significant changes occurred in all groups with thinning of the RPE layer and the decrease of the number of cells. IS/OS layer cytoplasm was lightly stained and swollen after daily exposure of 3 h (Figure 4(j)). In the daily exposure of 6 h group, IS/OS layer cytoplasm was sparse, lightly, swollen, and unevenly stained (Figure 4(k)). In the daily exposure of 12 h group, the IS/OS layer cytoplasm was very bright, and only a few were stained with cytoplasmic powder dyeing (Figure 4(l)). These tests were repeated 3 times for each rat, and there were 6 rats per group.

### 3.4. Long-Term Cumulative Blue Light Damages the Structure of the Retina

**Figure 5:**
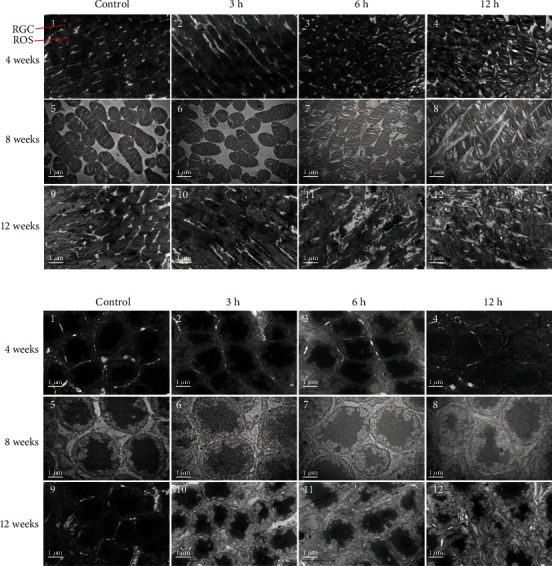
Ultrastructural images of the retinas of rats as observed by transmission electron microscopy. (a) Cross section for ultrastructural analysis of the retina of rats. (1, 5, and 9) The outer membrane disc of photoreceptors of rats in the normal control group was clear and complete, arranged orderly, with uniform chromatin and abundant cytoplasm; (2) the ultrastructure of retina of rats in the experimental group did not change significantly at 4 weeks 3 h; (3) the outer membrane disc of rats in the experimental group was loose at 4 weeks 6 h. (4) At 4 weeks 12 h, the membrane disc of the experimental group was loose, edema and a large number of vacuoles were formed, and the chromatin of the cell body was clearly thin; (6): at 8 weeks 3 h, the membrane disc of the outer segment of the experimental group was loose, and the chromatin of the cell body was thin; (7): at 8 weeks 6 h, the membrane disc of the outer segment of the experimental group was loose and disordered, vacuole was formed, and the chromatin of the cell body was thin, edge set; (8): at 8 weeks 12 h, the outer ganglion membrane disc was loose and edematous, and a large number of vacuoles were formed; the chromatin of the cell body was clearly diluted, and the organelles were reduced; (10): at 12 weeks 3 h, the outer ganglion membrane disc was loose and edematous, vacuoles were formed, and the membrane disc was destroyed; (11): at 12 weeks 6 h, the outer ganglion membrane disc was loose and showed edema, vacuole formation, and membrane disc destruction, the chromatin of the cell body became clearly light, and organelles decreased in size. (12) In the 12 h experimental group, there were a large number of vacuoles and structural damage in the disc of outer ganglion membrane, and the cell bodies were fuzzy, organelles were reduced, and the cytoplasm was dissolved. (b) Longitudinal ultrastructural section of the retina of rats. The scale bar is 12000 *μ*m. These tests were repeated 3 times for each rat, and there were 6 rats per group.

The discs of the external ganglion membrane of the photoreceptor in the control rats at 4, 8, and 12 weeks were clear and arranged in an orderly manner, and the chromatins were symmetrical and abundant (Figures 5(a), 1, 5, and 9 and 5(b), 1, 5, and 9). After 4 weeks of continuous irradiation, there was no obvious change in the ultrastructure of the retina after daily exposure 3 h (Figures 5(a), 2 and 5(b), 2). In the daily exposure 6 h group, the arrangement of the outer ganglion membrane disc was loose, a small number of vacuoles were formed, and the chromatin became light (Figures 5(a), 3 and 5(b), 3). In the daily exposure 12 h group, the membrane disc was loose and edematous, a large number of vacuoles were formed, and the chromatin became light (Figures 5(a), 4 and 5(b), 4). The outer ganglion disc was loose, and the chromatin became light after 8 weeks in the daily exposure 3 h group (Figures 5(a), 6 and 5(b), 6). In the daily exposure 6 h group, the outer ganglion disc was loose and disordered, vacuoles formed, the chromatin became light, and the edge gathered (Figures 5(a), 7 and 5(b), 7). In the daily exposure 12 h group, the outer ganglion disc was loose and edematous, a large number of vacuoles formed, the chromatin was clearly light, and the organelles were reduced (Figures 5(a), 8 and 5(b), 8). The outer ganglion membrane disc was loose, the chromatin became light, and the cytoplasm decreased after 12 weeks in daily exposure 3 h group (Figures 5(a), 10 and 5(b), 10). The outer ganglion membrane disc was loose and showed edema, vacuole formation, and membrane disc destruction, the chromatin became obviously light, and organelles decreased in size in the daily exposure 6 h group (Figures 5(a), 11 and 5(b), 11). The outer ganglion membrane disc contained many vacuoles, structural destruction, fuzzy cell bodies, decreased organelles, and dissolved cytoplasm after 12 weeks in the daily exposure 12 h (Figures 5(a), 12 and 5(b), 12). These tests were repeated 3 times for each rat, and there were 6 rats per group.

## 4. Discussion

In this study, the clinical investigation of the multifocal electroretinogram of the terminal video of medical staff was first conducted. The results showed that the daily use of the video terminal for more than 8 hours reduced the amplitude of the retinal photoreceptor cells in the parafoveal region of the macula and delayed the peak time. Then, we explored the effect of simulated mobile phone blue light (455 nm-470 nm, 5.03 lux, 0.123 W/m^2^) on the morphological structure and function of the optic nerve and retina of SD rats through animal experiments. SD rat retinal photoreceptor cells and pigment epithelial cells showed different degrees of degeneration. The longer the light exposure time and accumulation time of the rats, the more severe the damage to rat retinal photoreceptor cells. Blue light chronic photodamage mainly involved photoreceptor cells and ganglion cells of the retina. Chronic retinal photodamage mainly involves retinal photoreceptor cells, ganglion cells, bipolar cells, and Muller cells, which is positively correlated with time [16, 17]. The expression of retinal rhodopsin is reduced, and the longer the irradiation time, the more serious the damage.

Our experimental results found that in SD rats, the cytoplasm of the visual cells is transparent, and the RPE layer is thinned. The photoreceptor membrane disk is vacuolated, and the structure is destroyed. The A and B wave amplitudes of visual electrophysiological of dark adaptation to the environment are reduced, and the incubation period is prolonged. Long-term low-illumination blue light irradiation can cause rod cell damage in rats. The apoptosis of photoreceptors (including rod cells and cone cells) is the essential cause of blue light damage to the retina. Organisciak and Vaughan and other studies [18] [19] have found that after photoreceptor cell apoptosis, the outer membranous disc fall off, and the pigment epithelial cells are overwhelmed by phagocytosis and digestion, which will cause the atrophy of pigment epithelial layer and the destruction of the blood-retinal barrier.

There are two types of retinal light damage: one occurs under long-term low-level light exposure and is called the first type of photochemical damage [20]. The second type is the acute retinal light damage described by Ham et al. [21], which occurs under short-term high-intensity light exposure. This study belongs to the first type of light damage. This study found that low-illumination blue light caused different degrees of chronic damage to the retina, providing a reference for the rational use of mobile phones. Previous experimental studies have mostly focused on acute light damage caused by high-brightness white light [22, 23] and toxic levels of blue light [24], and these doses are much higher than our blue light dose. Chronic damage to blue light at low light levels is often overlooked because it is difficult to detect. This experiment simulates the degree of damage to human eyes caused by blue light in a low-brightness mobile phone screen. Because the spectrum emitted by the mobile phone screen is a composite spectrum, it is not possible to directly measure the blue light intensity. In this experiment, the QB4 colored glass filter that can pass through the blue wavelength is selected to filter the spectrum other than blue light [25], so as to measure the experimental mobile phone screen projection. Pure blue illuminance value at a distance from the normal viewing screen of the human eye is 5.03 lux (0.123 W/m^2^). This experimental method is unique and more practical and convincing than the study of high-illumination blue light irradiation. In this study, the low illumination chronic light injury animal model has good stability and repeatability, which provides a reference for further study on the prevention and treatment of chronic retinal light injury and retinal degeneration. No relevant reports have been seen before.

The results of H&E showed no change in 4 weeks 6 h and 12 h, while the results of electron microscope showed changes in the structure of retinal in 4 weeks 6 h and 12 h. This phenomenon may have the following three reasons. (I) The magnification of H&E is different from the magnification of electron microscope, and electron microscope can see more microscopic cell structure. (II) The retinal pigment epithelium (RPE) does not change, because it takes a certain amount of time to cause pathological changes in RPE. (III) The blue light radiation dose to the retina of freely moving rats depends on the posture of the head of the rat. In our experiment, we designed one of the rat's retinas is used for H&E and the other one for the electron microscope, so this may also cause slight differences between the H&E experimental results and the electron microscope results.

Due to the limitation of conditions, this experiment uses rats as the research object. Rats have no macula, but the RPE is consistent with humans. RPE cells are very sensitive to blue light stimulation, and blue light is absorbed by autofluorescent groups in RPE cells, which will trigger the cell and cause a series of cascade reactions, dysfunction, and even lead to cell death. Long-term accumulated blue light damage will induce or even accelerate macular degeneration of the retina [26, 27]. Thus, the rats meet the experimental conditions.

Rats and humans have different retina sizes, different diopters [28], and different density gradients of photoreceptor [29, 30]. Despite these differences between the rat and human retinas, the basic organization of the retina is conservative. The retinas of rats and humans are comparable to a certain extent [31]. Research showed an action spectrum matching the absorption spectrum of rhodopsin in rat and an action spectrum for photochemical damage from the UV to long visible wavelengths in macaque. Later, such a spectrum was also found in rat [32]. At the same time, our research found that the experimental results of rats are consistent with the results of clinical studies, and both will cause a decline in the function of photoreceptor cells. Studies have found that the diopter of humans is 165 folds (for the same luminance) than rats [28, 33], but our experimental results prove that the degree of damage to the retina of rats is not linearly related to the diopter. Our experimental results found that the rat retinas were loose and edematous in IS/OS layer in 4 weeks 6 h blue light irradiation. However, no significant pathological changes were seen in the IS/OS layer of the retina in 4 weeks 3 h. Therefore, this dose of blue light is not as high as 165 times for rats, but belongs to low-dose irradiation.

By comparing the results of rat experiments, it was found that the morphological and structural changes of the rat omentum were consistent with the functional changes, and the decline of the omentum function was accompanied by the morphological and structural degeneration. The chronic damage caused by blue light is worth noting.

## 5. Conclusions

The daily use of the video terminal for more than 8 hours reduced the amplitude of the retinal photoreceptor cells in the parafoveal region of the macula and delayed the peak time. Long-term exposure to low-illuminance blue light can cause retinal tissue structure and functional damage, and the chronic damage due to low-illuminance light warrants attention.

## Figures and Tables

**Table 1:** Mobile phone blue light illumination measurement.

Mobile phones brands	Illumination after filtration (lux)	Phone blue light illumination (lux)
A	2.44	4.34
B	3.18	5.65
C	3.18	5.65
D	2.83	5.03

This test was repeated not less than 6 times.

**Table 2:** Comparison of N1 and P1 wave amplitude density and peak time of Mf-ERG 2 ring and Mf-ERG 3 ring.

	Groups	*n*	N1 wave amplitude density		N1 peak		P1 wave amplitude density		P1 peak	
			Right eye	Left eye	Right eye	Left eye	Right eye	Left eye	Right eye	Left eye
Mf-ERG 2 ring	Observation group	11	40.16 ± 17.72	40.62 ± 15.40	24.80 ± 2.15	24.16 ± 2.78^##^	101.80 ± 16.08	109.66 ± 14.61	44.47 ± 2.33	45.19 ± 2.18^∗^^#^
	Control group	11	43.55 ± 11.06	46.32 ± 10.09	24.16 ± 2.02	25.41 ± 2.36	111.41 ± 22.10	122.56 ± 18.88	43.42 ± 1.64	42.78 ± 2.65
Mf-ERG 3 ring	Observation group	11	26.37 ± 9.58	21.60 ± 5.73^∗∗^	24.35 ± 1.50^∗^	24.16 ± 2.67	65.90 ± 15.38	67.41 ± 9.14	42.15 ± 2.32	43.49 ± 2.21^#^
	Control group	11	26.23 ± 5.47	27.94 ± 3.76	22.57 ± 2.22	23.46 ± 1.92	69.98 ± 11.95	74.06 ± 14.96	43.49 ± 1.59	43.75 ± 1.25

^∗^ *P* < 0.05 compared with the control group, ^∗∗^*P* < 0.01 compared with the control group, ^#^*P* < 0.05 compared with the right eye, and ^##^*P* < 0.01 compared with the right eye. These tests were repeated not less than 6 times.

**Table 3:** A wave results in photoreceptors of the retina.

	Control	4 w 3 h	4 w 6 h	4 w 12 h	8 w 3 h	8 w 6 h	8 w 12 h	12 w 3 h	12 w 6 h	12 w 12 h
Amplitude	146.17 ± 5.93	62.80 ± 3.62	67.45 ± 3.10	107.79 ± 24.28	124.73 ± 5.21	77.79 ± 0.60	108.87 ± 3.20	144.37 ± 5.56	127.72 ± 3.87	110.77 ± 6.67
Incubation period	71.42 ± 1.13	54.72 ± 2.16	54.47 ± 1.18	55.42 ± 0.92	79.35 ± 3.26	74.65 ± 1.87	80.08 ± 2.64	53.48 ± 1.35	70.27 ± 1.39	75.80 ± 1.76

These tests were repeated 3 times for each rat, and there were 6 rats per group.

**Table 4:** B wave results in retinal bipolar cells and Muller cells.

	Control	4 w 3 h	4 w 6 h	4 w 12 h	8 w 3 h	8 w 6 h	8 w 12 h	12 w 3 h	12 w 6 h	12 w 12 h
Amplitude	−5.74 ± 0.75	−11.82 ± 0.29	−7.78 ± 0.33	−25.43 ± 0.83	−14.96 ± 0.87	−11.45 ± 0.43	−5.25 ± 0.32	−11.23 ± 0.57	11.37 ± 0.77	−14.43 ± 0.91
Incubation period	12.45 ± 0.81	35.00 ± 2.59	17.68 ± 1.48	11.93 ± 0.95	23.60 ± 1.52	22.55 ± 0.83	23.58 ± 0.88	32.67 ± 0.67	30.35 ± 1.34	30.02 ± 1.74

These tests were repeated 3 times for each rat, and there were 6 rats per group.

**Table 5:** Statistical analysis of two factors and their interaction in the A wave and B wave.

	*Λ*		*F*		*ν*1		*ν*2		*P*	
	A	B	A	B	A	B	A	B	A	B
Irradiation time	0.00061729	0.00002866	210.96	998.63	16	16	86	86	<.0001	<.0001
Radiation dose	0.00022726	0.00003931	351.18	851.97	16	16	86	86	<.0001	<.0001
Time^∗^ dose	0.00000011	0.00000182	376.82	175.38	32	32	160.17	160.17	<.0001	<.0001

**Table 6:** Statistical analysis of the three treatment methods (irradiation time) of controlling dose factors in the A wave and B wave at different levels.

	*Λ*		*F*		*ν*1		*ν*2		*P*	
	A	B	A	B	A	B	A	B	A	B
3 h	0.00011415	0.0000029	92.60	583.27	16	16	16	16	<.0001	<.0001
6 h	0.0000017	0.0000005	759.72	1467.82	16	16	16	16	<.0001	<.0001
12 h	0.0000032	0.0000005	556.47	1467.82	16	16	16	16	<.0001	<.0001

These tests were repeated 3 times for each rat, and there were 6 rats per group.

## Data Availability

The datasets used and/or analyzed during the current study are available from the corresponding author on reasonable request.

## Supplementary Materials

Supplementary Materials Supplementary material related to the survey file for the daily use of video terminals in this article can be found in the online version.
