# Supplementary material for: Blue Light from Cell Phones Can Cause Chronic Retinal Light Injury: The Evidence from a Clinical Observational Study and a SD Rat Model
Source: Biomed Res Int. 2021 May 16;2021:3236892. doi: 10.1155/2021/3236892 (PMC8147535; doi:10.1155/2021/3236892)
Supplement: Supplementary Materials — Supplementary material related to the survey file for the daily use of video terminals in this article can be found in the online version. [file 3236892.f1.docx]

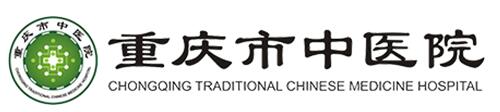


**Standardized scale of ophthalmic blue light subjects**

| ●The purpose of this questionnaire survey is to understand whether the long-term close use of mobile phones, computers and other video terminals will have adverse effects on the function and structure of the macular area, thus affecting vision, so as to provide reference for the correct and safe use of mobile phones  ●For those qualified for clinical enrollment (myopia ≤300 degrees, no organic eye disease, no hereditary eye disease, age between 20 and 40 years old), OCT, comprehensive optometry, visual acuity tests are required to understand the function of macula  ●This examination is a routine ophthalmic examination, safe and reliable  ●This scale is only used for scientific research purposes. Your personal information will be strictly protected. Your personal information will be hidden from the paper report |
| --- |

**Personal information**

| The name： gender： age： |
| --- |
| Native place：□Chongqing □Sichuan province □Other provinces and cities（Please indicate the）:____________ |
| Level of education：□Primary school □Junior high school □High school □Undergraduate course □A master's degree □Dr. |
| Professional：□Civil servants □Professional and Technical Personnel □ Workers □ Teachers □ Students □ Medical Workers □ Programmers □ Unemployed □ Others |

**Standardized scale**

| 1. What is the main video terminal you usually use?  □ Mobile phone □ Tablet computer □ Desktop computer □ TV □ Others |
| --- |
| 2. How long have you been using mobile phone, computer and other video terminals? (year)  1 2 3 4 5 6 7 8 9 10 others |
| 3. How much time do you spend on mobile phone and computer on average every day? (h)  1 2 3 4 5 6 7 8 9 10 11 12 others |
| 4. Do you feel uncomfortable after using mobile phone or computer for a long time? (pops)  □ dryness □ fatigue □ swelling pain □ foreign body sensation □ blurred vision□ photophobia □ tears □ envy □ others ________________ |
| 5. Do you usually use mobile phone in dark room and bright environment? Average time?  □Darkroom （hours） □Bright （hours） |
| 6. Do you have any history of eye surgery?  □No □Yes（Please specify the name of the operation）：________________ |
| 7. Do you have a history of eye trauma?  □No □Yes（Please indicate what kind of trauma）：­________________ |
| 8. Do you have a history of eye disease?  □No □Yes（Please indicate the disease）：­________________ |
| 9. What is your diopter?  □50° □100° □150° □200° □250° □300°  Others (Optometry Results) |
| 10. Have you ever taken any of the following special medications?  □Chloroquine □ Quinine □ Digitalis □ Hormones □ Antirheumatic □ Antimetabolites□ Antimalarial drugs □ Others：­­­_________ □I have not taken any of these drugs |
| 11. Have you ever had an eye examination for any of the following specialties?  □OCT □Visual acuity □ eye B-ultrasound □ visual field □ electrophysiology □ comprehensive optometry  □ Others: __________ □ I have not done any of the above |
| 12. Have you used any of the following protective measures when using electronic products?  □Indoor light □ wear protective glasses □ electronic products paste filter membrane □ anti-fatigue eye drops □ others :__________ □ I have not carried out the above protection |
| 13. How long do you think continuous use of mobile phones will not harm your eyes? (h)  □<1 hour □1-3 hours □>3 hours □ Others：__________ |
| 14. How do you think long-term use of mobile phones will affect the visual function?  □Vision loss □ tears □ photophobia □ shadow fluttering before eyes □ dryness □ double shadow□ Others ________ □ I don't think so |
| 15. Do you know what precautions can be taken to reduce the damage to eyes caused by electronic products?  □Reduce the time and frequency of use □ wear filter glasses □ take proper vitamins □ increase outdoor activities □ others: ________ □ I do not know these measures |
| 16 How often do you have regular eye examinations?  □Never □ rarely □ often (please specify every few months)：____________ |
|  |
|  |
|  |
|  |
|  |
|  |
|  |
|  |
